# Genome-wide association studies for citric and lactic acids in dairy sheep milk in a New Zealand flock

**DOI:** 10.1080/10495398.2024.2379897

**Published:** 2024-08-05

**Authors:** An Zongqi, Ana C. Marshall, J. M. D. R. Jayawardana, Mike Weeks, Simon M. Loveday, Warren McNabb, Nicolas Lopez-Villalobos

**Affiliations:** a Sichuan Agricultural University, College of Science and Technology, Farm Animal Genetic Resources Exploration and Innovation Key Laboratory of Sichuan Province, Chengdu, P. R. China; b School of Agriculture and Environment, Massey University, Palmerston North, New Zealand; c The Riddet Institute, Massey University, Palmerston North, New Zealand; d Department of Animal Science, Faculty of Animal Science and Export Agriculture, Uva Wellassa University, Badulla, Sri Lanka; e Smart Foods & Bioproducts Group, AgResearch Ltd, Massey University, Palmerston North, New Zealand

**Keywords:** Sheep milk, citric acid, lactic acid, genome-wide-association study

## Abstract

The objectives of this study were to estimate genetic parameters for citric acid content (CA) and lactic acid content (LA) in sheep milk and to identify the associated candidate genes in a New Zealand dairy sheep flock. Records from 165 ewes were used. Heritability estimates based on pedigree records for CA and LA were 0.65 and 0.33, respectively. The genetic and phenotypic correlations between CA and LA were strong-moderate and negative. Estimates of genomic heritability for CA and LA were also high (0.85, 0.51) and the genomic correlation between CA and LA was strongly negative (-0.96 ± 0.11). No significant associations were found at the Bonferroni level. However, one intragenic SNP in C1QTNF1 (chromosome 11) was associated with CA, at the chromosomal significance threshold. Another SNP associated with CA was intergenic (chromosome 15). For LA, the most notable SNP was intragenic in CYTH1 (chromosome 11), the other two SNPs were intragenic in MGAT5B and TIMP2 (chromosome 11), and four SNPs were intergenic (chromosomes 1 and 24). The functions of candidate genes indicate that CA and LA could potentially be used as biomarkers for energy balance and clinical mastitis. Further research is recommended to validate the present results.

## Introduction

Sheep dairy farming is growing in New Zealand, and some dairy cattle farms in the North Island have recently shifted to dairy sheep farms.^1^ New Zealand accounted for 0.1% of the total sheep milk production worldwide until 2020.^2^ Sheep milk has a high content of protein (as well as casein), fat, lactose, vitamins, and minerals,^3^ which makes it ideal for processing, particularly into cheese.^4^ Sheep milk is also a richer source of unsaturated fatty acids (UFA) when compared to cow and goat milk.^5^ Moreover, the bioactive substances present in sheep milk, such as organic acids, prebiotics, and probiotics, can be used for the production of cosmetics and for promoting human health.^6^

Citric acid or citrate (CA) and lactic acid (LA) are considered anti-ageing factors^7^ and are some of the least studied components in sheep milk. Sheep milk has been reported to have a higher content of CA and LA than goat milk,^8^^,^^9^ but lower CA content than cow milk.^10^ Citric acid is one of the main metabolites synthesized in the mammary gland, whose carbon is derived from glucose and acetate.^11^^,^^12^ Formed within the secretary cell, CA enters milk by exocytosis from the Golgi vesicles, in a similar way to lactose and protein.^12^ Approximately 7% of total CA in cow milk is in the non-aqueous phase, combined with calcium-casein complexes,^13^ but the majority of CA in milk is soluble.^14^ Furthermore, CA is an intermediate in the tricarboxylic acid cycle, also known as the Krebs cycle, which is one step of cellular metabolism to generate energy.^15^ Citric acid is also indirectly involved in fat synthesis by providing the reducing equivalents in the form of nicotinamide adenine dinucleotide phosphate (NADPH).^15^

The CA content has been proposed to be used as an indicator of the energy status of dairy cows.^16^ A higher amount of CA in the milk was found in cows with negative energy balance (NEB). When the energy requirements for maintenance and milk production are not satisfied by feed intake, the cow enters a status of NEB, especially in early lactation when the energy requirement for milk production is at its highest peak.^17^ It has been suggested that increases in the lipid metabolism of mammary gland cells affect the milk content of CA in cows with negative energy balance. The reduced levels of CA from mid-lactation have been suggested to be due to improved energy balance.^16^

This is further supported by the association of CA content with the synthesis of fatty acids. Citrate concentration in milk was increased when de novo synthesis of fatty acids in the mammary cells increased. The changes in CA content in milk were related to changes in intra-mammary energy metabolism and the level of fatty acid synthesis.^15^

Citric acid is also known to influence the technological properties of milk. Citric acid content in milk has been linked with milk heat stability, which is relevant in the processing of milk into dairy products that require high-temperature treatment (such as UHT, and milk powder). A deficiency of citrate leads to an excess of soluble calcium, which destabilizes the casein micelles.^18^ The effect of CA on cheesemaking has also been investigated.^19^^,^^20^ Increased citrate levels in skim milk from individual cows have previously been associated with impaired milk coagulation properties for cheesemaking,^19^ but the opposite effect has also been documented with an improvement of milk coagulation with a higher CA.^20^ The concentration of CA varies not only between breeds of dairy cattle,^20–22^ but also between animals of different genotypes. For example, a high level of CA was shown for animals carrying the A1 allele type of β-casein,^23^ and a negative correlation between CA and fat percentage in cow milk has been reported.^21^ Estimates of heritability for CA were reported to be high; 0.54 to 0.82.^20^^,^^21^

Lactic acid, on the other hand, is the main organic acid representing titratable acidity in milk^24^ and is mainly produced by lactic acid bacteria (LAB) breaking down lactose,^25^ which means that fresh milk contains very low LA (not over 0.002%).^26^ Furthermore, the estimate of heritability for LA has been previously reported to be close to zero.^21^ Lactic acid produced by bacterial carbohydrate fermentation contributes to acidification and flavor development in cheesemaking. An increase of 0.03% of LA content in milk has been related to a protein content increase of over 1%.^27^

Contents of CA and LA have been proposed as metabolite biomarkers for the health status of dairy animals and for mammary gland disease due to the correlation with somatic cell count (SCC) and therefore, with clinical mastitis.^28–31^ Additionally, the correlations between milk composition (fat, protein, and lactose) and these metabolites could have a wide range of applications in food processing.^28^

The genomic background of these traits has yet to be explored in dairy sheep, while investigations have only recently begun in dairy cows, making this study′s contribution noteworthy. In addition, performing GWAS in dairy sheep presents unique challenges compared to dairy cows. Sheep typically produce smaller volumes of milk, making milk sampling more difficult. Additionally, most dairy sheep farms lack milk meters due to their high costs relative to the value of a sheep.

Considering the above investigations and possible applications of CA and LA, the objective of this study was to estimate variance components, genetic and genomic parameters of CA and LA content in sheep milk and perform genome-wide analysis to identify SNPs associated with genes potentially affecting the concentration of CA and LA in dairy sheep milk.

## Materials and methods

### Animals and milk samples

This study was conducted at a commercial sheep dairy farm in Masterton, New Zealand (latitude: −40.9597, longitude: 175.6575). Animal ethics approval was obtained for this study (Massey University Animal Ethics Committee-Protocol 21/45). Detailed information on the flock is provided in.^32^ The age structure of the flock was 15% first-, 28% second-, 23% third- and 34% ≥fourth-parity ewes. The median lambing date of the flock was the 20^th^ of August 2021. Collections of test-day records started after the lambs were fully weaned. A total of 521 test-day records from once-a-day milking were gathered from 1 November 2021 until 31 January 2022 from 169 ewes.^32^ At least 2 milk tests were performed for each ewe, and 92% of the flock had at least 3 milk test records. On each test-day, milk volume was measured, and a representative milk sample was taken from each animal. The composition of individual milk samples was analyzed using a Fourier-transform Infrared (FTIR) milk-analyser (MilkoScan FT6000) calibrated for sheep milk components. The following milk components were extracted: percentages of fat, protein, lactose, total milk solids, and contents of CA and LA. Information on lambing date, litter size, and age of animals were supplied to the study.

### Genotyping and quality control

Animals were genotyped using the OvineSNP50 Beadchip array (Illumina, San Diego, CA) with a medium-density SNP panel (50k SNPs). Ear tissue samples from 323 ewes (including the 169 ewes and 154 ewe lambs) and 6 rams were collected for DNA extraction and scanning using an iScan^®^ at the Equine Parentage Testing Lab (Massey University, Palmerston North). A total of 64,734 SNPs were obtained for quality control using the SNP & Variation Suite (SVS 8.8)^33^ software. In the filtering process, genomic records were removed for 23 animals with a call rate < 95% across all the SNPs, from which 4 animals were ewes that had phenotype records. Also, SNPs with > 1% missing genotypes across all individuals (call rate < 95%), and that had a significant deviation from the Hardy-Weinberg equilibrium threshold of p < $10^{-6}$ or that had minor allele frequency < 1% were also removed. After these quality control edits, a total of 306 animals with SNPs (including 165 ewes with phenotypes), and 45,801 SNPs remained for association analyses.

### Statistical analysis

Descriptive statistics were obtained using the MEANS procedure of SAS 9.4.^34^ Analysis of variance for concentration of CA and LA was performed using the MIXED procedure with a linear model that included the fixed effects of ewe coat color (black or white) as an indicator of variety within the breed, litter size (1 or ≥2 lambs), parity number (1st, 2nd, 3rd or ≥4th parity), milking month (Nov, Dec or Jan), and days in milk as a covariate, and the random effect of ewe to account for repeated records on the same ewe. Least-squares means for each class of fixed effects and standard errors were obtained and used for mean comparisons using Fisher’s least significant difference test. Lactation curves of CA and LA contents for the flock were modeled with a quadratic and a linear polynomial, respectively. The best order of the polynomial was based on the R^2^ values.

### Estimation of genetic parameters

ASReml release 4.1^35^ was used to estimate the variance components, heritability, and genetic and phenotypic correlations between CA and LA. Heritability estimates for CA and LA were obtained through single-trait animal models, and estimates of genetic correlation were obtained through a bivariate animal model. The single-trait animal model was represented as follows:

y=Xb+Za+Wc+e

where **y** is the vector of phenotypic traits; **b** is the vector of fixed effects of ewe coat, litter size, parity, milking month, and days in milk as covariate; **a** is the vector of animal genetic effects; c is the vector of permanent (ewe) environment effect, e was the vector of random residual effects; **X, Z** and **W** are the design matrices relating to the records of fixed, additive genetic, and permanent environment effects, respectively.

The distributional properties of this model are the following:

$$E\left[\begin{array}{c} y\\ a\\ c\\ e \end{array}]=[\begin{array}{c} \text{Xb}\\ 0\\ 0\\ 0 \end{array}\;\right]$$

$$\text{var}\left[\begin{array}{c} a\\ c\\ e \end{array}\right]=\left[\begin{array}{ccc} A\sigma_{a}^{2} & 0 & 0\\ 0 & I_{1}\sigma_{c}^{2} & 0\\ 0 & 0 & I_{2}\sigma_{e}^{2}\; \end{array}\right]$$

where **A** is the numerator relationship matrix between all the animals in the pedigree, **I**_1_ is an identity matrix of order equal to the number of ewes with records, **I**_2_ is an identity matrix of order equal to the number of records, $\sigma_{a}^{2}$ is the animal genetic additive variance, $\sigma_{c}^{2}$ is the permanent environment effect variance and $\sigma_{e}^{2}$ is residual error variance. The pedigree file included 165 animals with records, which were progeny of 21 sires and 105 dams, with 11 maternal grandsires and 25 maternal granddams.

The heritability ($h^{2}$) estimate was obtained as follows:^36^

$$h^{2}=\frac{\sigma_{a}^{2}}{\sigma_{a}^{2}+\sigma_{c}^{2}+\sigma_{e}^{2}}\;$$

The repeatability (t) estimate was obtained as follows:^36^

$$t=\frac{\sigma_{a}^{2}+\sigma_{c}^{2}}{\sigma_{a}^{2}+\sigma_{c}^{2}+\sigma_{e}^{2}}\;$$

The bivariate model was represented in matrix notation, as follows:

$$\left[\;\begin{array}{c} \;y_{1}\\ y_{2} \end{array}]=[\begin{array}{cc} X_{1} & 0\\ 0 & X_{2}\; \end{array}\;\right]\left[\;\begin{array}{c} b_{1\;}\\ b_{2} \end{array}]+[\begin{array}{cc} \;Z_{1} & 0\\ 0 & Z_{2}\; \end{array}\right]\left[\begin{array}{c} \;a_{1}\;\\ a_{2} \end{array}]+[\begin{array}{cc} \;W_{1} & 0\\ 0 & W_{2}\; \end{array}\right]\left[\begin{array}{c} \;c_{1}\;\\ c_{2} \end{array}]+[\begin{array}{c} e_{1}\;\\ e_{2} \end{array}\;\right]$$

where ${\text{ y}}_{1}$ and ${\text{ y}}_{2}$ are the vectors of phenotypic measures of CA and LA; $b_{1\;}$ and $b_{2\;}$ are the vectors of fixed effect of coat color, litter size, age, milking month, linear and quadratic effects of days in milk; $X_{1}$, $X_{2}$, $Z_{1}$, ${\;Z}_{2}$**, W_1_** and **W_2_** are design matrices relating the fixed, animal additive genetic and permanent environment effects related to the $y_{1}$ and $y_{2}$ phenotypes, respectively, $a_{1}$ and $a_{2}$ are the vectors of random effects of animal for each trait; **c_1_** and **c_2_** are the vectors of random permanent environment effects, and $e_{1}$ and $e_{2}$ are vectors of residual errors for each trait. The expected values of the variables were assumed **E(**$y_{1}$**) =** $X_{1}b_{1}$; **E(**$y_{2}$**) =** $X_{2}b_{2}$ with variances and covariances of random effects as follows:

$$\text{var}[\begin{array}{c} \begin{array}{c} a_{1}\\ a_{2}\\ c_{1}\\ c_{2} \end{array}\\ e_{1}\\ e_{2} \end{array}]=[\begin{array}{cccccc} A\sigma_{a1}^{2} & A\sigma_{a12} & 0 & 0 & 0 & 0\\ A\sigma_{a12} & A\sigma_{a2}^{2} & 0 & 0 & 0 & 0\\ 0 & 0 & I_{1}\sigma_{c1}^{2} & I_{1}\sigma_{c12} & 0 & 0\\ 0 & 0 & I_{1}\sigma_{c12} & I_{1}\sigma_{c2}^{2} & 0 & 0\\ 0 & 0 & 0 & 0 & {I_{2}\sigma }_{e1}^{2} & I_{2}\sigma_{e12}\\ 0 & 0 & 0 & 0 & I_{2}\sigma_{e12} & I_{2}\sigma_{e2}^{2} \end{array}]$$

where $\sigma_{a1}^{2}$ is the additive genetic variance of the CA content, $\sigma_{a2}^{2}$ is the additive genetic variance of LA content and $\sigma_{a12}$ is the additive genetic covariance between CA and LA contents; $\sigma_{c1}^{2}$ is the permanent environment variance of the CA content, $\sigma_{c2}^{2}$ is the permanent environment variance of LA content and $\sigma_{c12}$ is the permanent environment covariance between CA and LA contents; $\sigma_{e1}^{2}$ is the residual variance for CA content; $\sigma_{e2}^{2}$ is the residual variance of LA content and $\sigma_{e12}$ is the residual covariance between CA and LA contents.

The genetic correlation ($r_{g}$) between CA and LA was estimated as follows:^36^

$$r_{g}=\frac{\sigma_{a12}}{\sigma_{a1}\times \sigma_{a2}}$$

where $\sigma_{a1}$ and $\sigma_{a2}$ are genetic additive standard deviations for CA and LA contents, respectively.

The phenotypic correlations ($r_{P}$) were estimated as follows:^36^

$$r_{P}=\frac{\sigma_{p12}}{\sigma_{p1}\times \sigma_{p2}}$$

where $\sigma_{p1}$ and $\sigma_{p2}$ are the phenotypic standard deviation for CA and LA contents, respectively, and $\sigma_{P12}$ was the phenotypic covariance between CA and LA contents, with $\sigma_{p1}^{2}=\sigma_{a1}^{2}+\sigma_{c1}^{2}+\sigma_{e1}^{2}$ and $\sigma_{p2}^{2}=\sigma_{a2}^{2}+\sigma_{c2}^{2}+\sigma_{e2}^{2}$.

### Genomic-wide association analysis

The phenotypic traits of CA and LA were pre-corrected for fixed effects using the single-trait animal repeatability model in the ASReml 4.1 software package.^35^ The models included the fixed effects of coat color, litter size, age, milking month, linear and quadratic effects of days in milk as covariates, the random effect of ewe permanent environment, and random residual. Principal component analyses for population stratification correction were performed. PC1 was highly correlated (r = 0.95) with the coat-color effect and therefore PCs were not included, instead, the genomic relationship matrix was used for adjustment of population structure. The genome-wide association study (GWAS) was performed using a mixed linear model in the software package.^37^ The following model was fitted for each trait:

y=μ+Xβ+g+e

where y was the vector of adjusted phenotype for each ewe, μ was the vector of overall mean, **β** was the vector of fixed effects and **X** was the incidence matrix of β to y with the SNPs genotypes of BB, AB, or AA, respectively, g was the random additive polygenic effect and e was the random residual error, The assumptions for the model were: $g∼N\left(0,G\sigma_{g}^{2}\right)$, where **G** was the genomic relationship matrix between the ewe and $\sigma_{g}^{2}$ was the additive genetic variance explained by SNPs, and $e∼N\left(0,I\sigma_{e}^{2}\right)$, where **I** was the identity matrix of order n = 165 and $\sigma_{e}^{2}$ was the residual variance.

Bonferroni multiple-test correction and the suggestive significance threshold were used for multiple tests, which is considered conservative, to avoid the type I error.^38^ Bonferroni multiple-test correction was estimated at 5% genome-wide significance as 0.05/m (0.05/45,801 = 1.09 ×$10^{-6}$), which corresponds to 5.96 on a − log10 (p-value) scale. The suggestive significance threshold was estimated at 1/m (1/45,801 = 2.18×$10^{-5}$), which was 4.66 on a − log10 (p-value) scale. The chromosomal significance threshold was calculated as 0.05/number of SNPs in each chromosome on a − log10 (p-value) scale. Manhattan plots in which − log10 (p-values) were plotted against their genomic locations of the markers for each trait using the qqman package in R software 4.2.1.^39^

### Candidate genes and functional analysis

Ensembl Release 109, based on the Ovis aries (sheep) reference genome in Oar_rambouillet_v1.0 genome assembly (http://www.ensembl.org/index.html, accessed on Feb 2023), was used to identify potential candidate genes in association analysis.^40^ Gene annotation boundaries were set at 150 kbp on either side of significantly associated SNP. This range was chosen due to the density of the chip array. The biological functions of the associated candidate genes were reviewed using the Gene Ontology (GO) tool in Ensembl.

For LA candidate genes, enrichment of gene ontology (GO) was investigated using the STRING Genomics 10.0 database.^41^ This analysis of protein-protein interaction was based on text mining, experiments, database, co-expression, neighborhood, gene fusion, and co-occurrence, and the minimum required interaction score was set to 0.40 and with a false discovery rate (FDR) <0.05.

## Results

### Descriptive statistics

Descriptive statistics of milk components and CA and LA contents for dairy sheep in the 2021–2022 season (n = 165 individuals) are presented in Table 1 and data distribution plots for CA and LA contents are presented in Figures S1 and S2.

**Figure 1.**
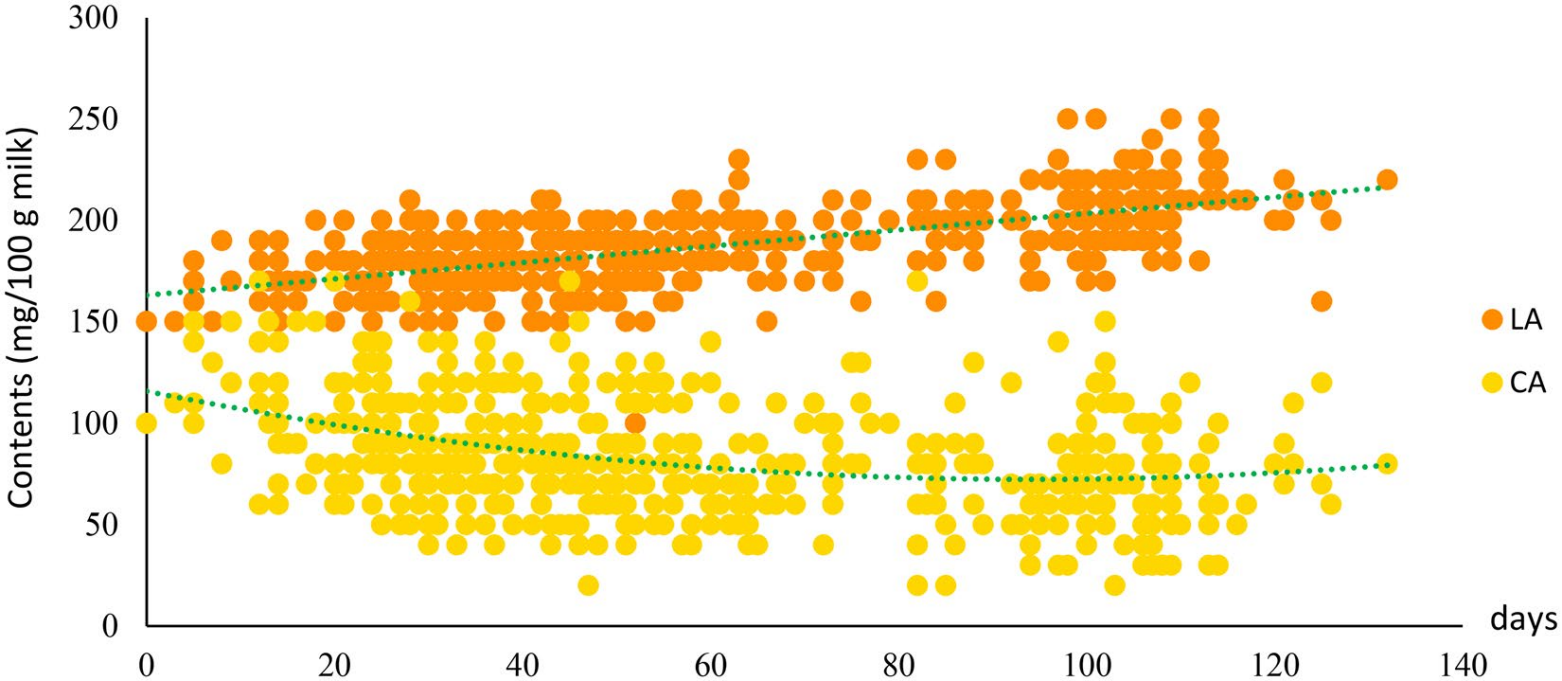
Variation of citric acid (CA; mg/100 g milk) and lactic acid (LA; mg/100 g milk) contents throughout the study period. Linear and quadratic models were used for LA and CA, respectively; days = days in milk, was presented by days in lactation minus 50.

**Figure 2.**
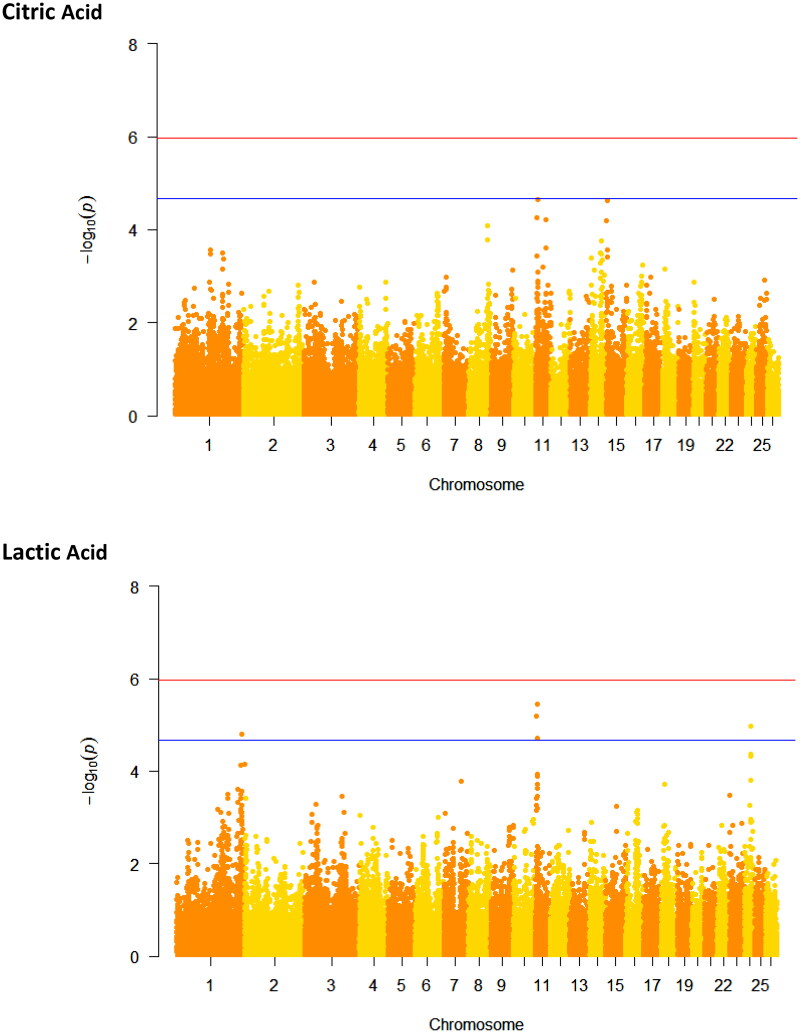
Manhattan plots for − log10 (p-values) of marker effects for citric acid and lactic acid in dairy sheep milk. The genome-wide significance threshold of Bonferroni correction is represented by the red line at − log10 (p-value) = 5.96, and the suggestive significance threshold is represented by the blue line at − log10 (p-value) = 4.66.

**Table 1.** Descriptive statistics^1^ of milk components including citric and lactic acids in dairy sheep sampled during the production season 2021-2022 in a commercial flock in New Zealand (N = 622 test-day milk samples from 165 ewes).

Trait	Mean	SD	Min.	Max.	CV (%)
Fat, %	6.15	0.69	4.23	8.55	11
Protein, %	5.40	0.44	4.31	6.98	8
Lactose, %	4.76	0.16	4.00	5.13	3
Total milk solids, %	16.9	0.96	14.2	20.0	6
Citric acid, mg/100 g	83.7	29.0	20.0	170.0	35
Lactic acid, mg/100 g	186.9	20.2	140.0	250.0	11

^1^ SD = standard deviation, Min. = minimum value, Max. = maximum value and CV = coefficient of variation.

Ewes produced milk with high percentage of fat (6.15%) followed by protein (5.40%) and lactose (4.76%) percentages during the production season. The content of LA was higher than the content of CA, but LA had a smaller coefficient of variation. The lactation curves for LA and CA are shown in Figure 1. Throughout the lactation, LA content slowly increased, whereas CA content decreased during the first 70 days, and then stabilized, rising slowly until the end of the study period.

### Factors affecting the contents of citric and lactic acids

Least-squares means and standard errors of LA and CA for fixed effects of coat color, age, litter size, and milking month are presented in Table 2.

**Table 2.** Least-squares means, standard errors (SE), and p-value for fixed effects on contents of citric acid (CA; mg/100 g milk) and lactic acid (LA; mg/100 g milk) in milk from ewes (n = 165) of different coat colors, parities, litter sizes and milking months.

Effect	Citric acid			Lactic acid		
	Mean	SE	p-value	Mean	SE	p-value
Coat color			0.05			0.79
Black	76.1	4.4		184.1	2.4	
White	85.8	2.4		184.8	1.2	
Litter size			0.41			0.10
1	82.6	3.3		182.6	1.8	
2	79.3	3.2		186.3	1.7	
Parity			0.16			0.05
1	85.4	5.8		180.7	3.2	
2	76.9	4.1		188.9	2.2	
3	76.2	4.1		182.5	2.3	
≥4	85.2	3.4		185.8	1.9	
Lactation month			0.16			<0.001
November	84.3	3.8		181.0^b^	2.2	
December	78.8	3.1		178.6^b^	1.8	
January	79.6	3.5		193.8^a^	2.0	

^a,b^Means with different superscripts within effect are significantly different (*p* < 0.05).

The milking month of ewes was significant (*p* < 0.01) for LA concentration and there was no significant effect on these fixed effects for CA composition. However, the coat color and ewe age had nearly significant effects on the CA and LA content, respectively.

### Genetic parameters for contents of citric and lactic acids

Table 3 shows the variances and heritabilities for CA and LA, and genetic and phenotypic correlations between CA and LA, using pedigree-based variance and genomic variance components.

**Table 3.** Estimates of variance components and heritabilities with their respective standard errors (in brackets) were obtained from pedigree-based analysis considering the additive genetic relationship matrix and genomic-based analysis considering the genomic relationship matrix for citric acid (CA) and lactic acid (LA).

Pedigree-based variance component estimates^1^								
Trait	$\sigma_{a}^{2}$	$\sigma_{c}^{2}$	$\sigma_{e}^{2}$	$\sigma_{p}^{2}$	$h^{2}$	t	Correlations	
CA	511 (71)	0.0 (0.0)	276 (22)	786 (72)	0.65 (0.04)	0.65 (0.04)		$r_{p}$ = −0.40 (0.05)
LA	83 (57)	53 (48)	115 (9)	252 (23)	0.33 (0.21)	0.54 (0.05)	$r_{g}$ = −0.67 (0.33)	
Genomic-based variance component estimates^1^								
Trait	$\sigma_{a}^{2}$	-	$\sigma_{e}^{2}$	$\sigma_{p}^{2}$	$h^{2}$		Correlations	
CA	360 (90)	–	0.06 (0.05)	0.42 (0.06)	0.85 (0.13)			–
LA	50 (20)	–	0.05 (0.02)	0.11 (0.01)	0.51 (0.17)		$r_{g}$ = −0.96 (0.11)	

^1^All variance components correspond to contents of CA and LA (mg/100 g of milk): $\sigma_{a}^{2}$ = additive genetic variance; $\sigma_{e}^{2}$ = residual variance; $\sigma_{c}^{2}$ = permanent environment variance; $\sigma_{p}^{2}$ = total phenotypic variance; $h^{2}$ = heritability; t = repeatability; $r_{g}$ = genetic correlation between CA and LA; $r_{p}$ = phenotypic correlation between CA and LA.

Higher heritability values were found for CA than for LA using both genomic- and pedigree-based approaches. A strong negative genomic correlation (-0.96 ± 0.11) between CA and LA was found in this study, whereas the pedigree-based genetic correlation between CA and LA was strong-moderate (-0.67 ± 0.33).

### Genome-wide association studies for contents of citric and lactic acids

The Manhattan plots for CA and LA in dairy sheep milk are presented in Figure 2. The QQ-plots of CA and LA before and after data correction for stratification are presented in a supplementary file in Figures S3–S6.

No genome-wide significant associations were found for LA or CA contents in this population using the significance threshold of Bonferroni. However, at the suggestive threshold, a total of five SNPs were significantly associated with LA content.

### Candidate genes and functional analysis

The significant SNPs with respective candidate genes and gene annotation for CA and LA contents are presented in Tables 4 and 5, respectively. The gene ontology functional enrichment results for LA content are presented in Table 6.

**Table 4.** The single nucleotide polymorphisms (SNPs) were identified as significant at the chromosomal significance threshold (0.05/number of SNPs in chr) for citric acid (CA) content in dairy sheep milk.

SNP	Chr	Chr − log10 (p)	Position	Ref./ MA	Ref. Freq.	Effect (SE)	−log10 (p)	Annotation	Candidate genes^1^
OAR11_9907480.1	11	4.34	9907480	A/G	838.93	−14.42 (3.40)	4.65	Protein coding	**C1QTNF1**, TIMP2, LGALS3BP, CANT1, ENGASE, RBFOX3
OAR15_3892898.1	15	4.47	3892898	A/G	373.33	−10.38 (2.46)	4.62	Intergenic	ENSOARG00020014135

Chr = chromosome; Chr − log10 (p)= chromosomal significance threshold; Ref. = reference allele; MA = minor allele; Ref. freq. = reference allele frequency; SE = standard error. Reference allele frequency, effect and standard error presented were multiplied by 1000.^1^Candidate genes include those within 150 kbp distance either side of the significant SNP. Gene names marked in bold are those that include the intragenic SNP. *Closest gene within 5kbp distance either side of the significant SNP.

**Table 5.** The single nucleotide polymorphisms (SNPs) were identified as significant at the suggestive significance threshold (−log_10_ (*p*-value) ≥ 4.66) and/or at the chromosomal significance threshold (0.05/number of SNPs in chr) for lactic acid (LA) content in dairy sheep milk.

SNP	Chr	Chr − log10 (p)	Position	Ref./ MA	Ref. Freq.	Effect (SE)	−log10 (p)	Annotation	Candidate genes^1^
s64611.1	1	5.01	284089015	A/G	260.42	6.26 (1.45)	4.81	Intergenic	ENSOARG00020002543
s25858.1	11	4.34	9641214	A/G	690.00	6.01 (1.30)	**5.44**	Intron	**CYTH1**, DNAH17, TIMP2, ENSOARG00020011905
s56469.1	11	4.34	8356745	A/C	310.00	−5.84 (1.29)	**5.19**	Intron	**MGAT5B**, MFSD11, METTL23, SRSF2, JMJD6 ENSOARG00020016717
OAR11_9760146.1	11	4.34	9760146	A/G	630.87	5.50 (1.29)	**4.71**	Intron	**TIMP2**, ENSOARG00020011905, CYTH1, CANT1, LGALS3BP, C1QTNF1
s28518.1	24	4.11	26968037	A/G	676.67	−6.97 (1.58)	**4.98**	Intergenic	MAZ*, CDIPT, C16ORF54, KIF22, ZG16, QPRT, ENSOARG00020020033, ENSOARG00020019988, TAOK2, KCTD13, SEZ6L2, MVP, PAGR1, PRRT2, TMEM219, ENSOARG00020016749, ENSOARG00020019032, ENSOARG00020019335
OAR24_26621114.1	24	4.11	26621114	G/A	653.33	−6.12 (1.50)	**4.36**	Intergenic	NUPR1*, TUFM, ATP2A1, SH2B1, ATXN2L, CLN3, IL27, EIF3C, ENSOARG00020026028, ENSOARG00020026072, SGF29, MAPK3, GDPD3, YPEL3, TBX6, ALDOA, CORO1A, PPP4C, ENSOARG00020014726, ENSOARG00020014644, ENSOARG00020014591
OAR24_25044819.1	24	4.11	25044819	G/A	613.33	−5.50 (1.35)	**4.33**	Intergenic	–

Chr = chromosome; Chr − log10 (p) = chromosomal significance threshold; Ref. = reference allele; MA = minor allele; Ref. freq. = reference allele frequency; SE = standard error. Reference allele frequency, effect and standard error presented were multiplied by 1000. −log10 (p)=SNP significance, marked in bold if significant at the chromosomal significance threshold. ^1^Candidate genes include those within 150 kbp distance either side of the significant SNP. Gene names marked in bold are those that include the intragenic SNP. *Closest gene within 5kbp distance either side of the significant SNP.

**Table 6.** Gene Ontology (GO) functional enrichment for candidate genes for lactic acid content, with false discovery rate (FDR) <0.05.

Trait	Gene Ontology	Biological process description	Strength	FDR
LA	GO:0018279	Protein N-linked glycosylation via asparagine	2.17	0.0012
	GO:006487	Protein N-linked glycosylation	2.03	1.73e-11
	GO:006486	Protein glycosylation	1.72	3.50e-16
	GO:009101	Glycoprotein biosynthetic process	1.67	9.48e-17

Two SNPs significant at the chromosomal significance threshold, and seven associated candidate genes, located in the suggested region, were identified for CA content, in chromosomes 11 and 15. The most relevant SNP for CA was within gene C1QTNF1 in chromosome 11, in a protein-coding region. The second most relevant SNP was intergenic and close to the novel gene ENSOARG00020014135.

Seven SNPs and 53 associated candidate genes located in the suggested region were identified for LA content, in chromosomes 1, 11, and 24. Three SNPs out of seven were mapped to introns. Five out of seven SNPs were significantly associated at the suggestive significance threshold (−log_10_ (*p*-value) ≥ 4.66). Six SNPs out of seven were significant at the chromosomal significance threshold. Four out of seven SNPs were significant at both suggestive and chromosomal significance thresholds. The most significant SNP for LA content was in chromosome 11, an intron variant of gene cytohesin 1 (CYTH1). The other two SNPs in chromosome 11 were intron variants of the genes MGAT5B and TIMP2. Another strong SNP for LA content, significant at the chromosomal threshold, was intergenic in chromosome 24, close to gene MAZ. The gene C1QTNF1 was a common gene for CA and LA contents in the suggested region.

## Discussion

### Content and genetic parameters of citric and lactic acids

The average citric acid content in this flock was 83.7 mg/100 g milk. A study from Austria reported a higher CA content (154 ± 25 g/100 g milk).^9^ Contents of CA in dairy goat milk were reported at 102 ± 11 mg/100 g milk.^9^ The content of CA in cow milk (11.4 ± 0.4 mmoles/kg) was greater than in sheep milk (10.7 ± 1.4 mmoles/kg) and goat milk (4.2 ± 0.7 mmoles/kg).^10^ In Danish Jersey cows’ milk, the CA content was higher than in Danish Holstein′s milk,^20^ whereas in China, the LA content was higher in Holstein′s milk than in Jersey′s milk.^22^ In sheep milk, a significant difference in LA content was reported for Lacaune sheep raised in different regions of south Brazil.^24^ LA concentration in Lacaune sheep milk (240 mg/100 g milk) was higher than the LA content estimated in our study. A New Zealand study^8^ reported that sheep milk had a higher content of LA (220 to 250 mg/100 g milk) than cow milk (150 to 180 mg/100 g milk) and goat milk (140 to 230 mg/100 g milk).

Reports on the estimates of genetic parameters for CA and LA in sheep milk are scarce in the literature. However, heritability estimates for CA content in Danish Holstein (0.59 ± 0.20) and in Danish Jersey (0.82 ± 0.31)^20^ were greater than our estimates. Heritability for LA reported in the literature was reported to be close to zero,^21^ which contradicts the present reported heritability values for LA. The estimates of genetic and genomic correlations between CA and LA contents estimated in our study were strongly negative. This indicates that ewes with genetic merit for producing milk with higher CA content are of low genetic merit for LA content in milk. It is probably related to the co-metabolism of CA and glucose, which starts with the degradation of CA to pyruvate, generating LA as the end-product of glucose metabolism.^42^ Also, lactic acid bacteria produce LA from lactose and diacetyl from CA.^42^^,^^43^

In the present study, an opposite trend was observed between CA content and LA content throughout the lactation of dairy sheep. Citric acid content decreased in earlier stages and increased slowly until the end of lactation, agreeing with the previous study for cow and sow.^10^ Also, energy balance has been negatively correlated with milk fat yield (r = −0.78) in dairy cows, and CA yield has been positively correlated with fat yield throughout the lactation (r = 0.53), indicating that citrate is used for milk fat synthesis in cows in negative energy balance and therefore, could be used as an indicator of energy status.^15^^,^^16^^,^^44^ The underlying context of this is that the energy deficit leads to increased lipolysis, and the uptake of fatty acids mobilized from body fat tissue is increased resulting in a slowly increased fat synthesis in the mammary cells.^16^^,^^45^^,^^46^ Samples in our study were collected from around 50 days of lactation (the first 50 days were the lamb suckling period), thus the elevated content that is generally reported in the first 2-6 weeks could not be evaluated in our study.

Citric acid and LA have also been proposed as metabolic biomarkers for mastitis, with increased LA content and decreased CA content in clinical mastitis.^28–31^ Records on clinical mastitis were not available in this study and it was not possible to explore this relationship.

### Genome-wide association for the content of citric acid

The QQ-plots showed a proper fit for stratification and a deviation to the right which suggests a true association between SNPs and concentration of CA and LA.

In agreement with previous findings of for cow milk,^47^ a genomic locus in chromosome 11 was associated with CA content. However, studies in cow milk have also revealed strong candidate genes associated with CA content in bovine chromosome 20.^47^^,^^48^

The specific biological functions of the gene C1q and TNF Related 1 (C1QTNF1) are mainly related to the regulation of glucose metabolic processes (GO:0010906) and positive regulation of the MAPK cascade (GO:0043410). The gene C1QTNF1 participates in lipid metabolism as a novel adipokine.^49^ The increased uptake of glucose leads to accelerated production of citrate, and citrate is then converted by ACLY to acetyl-CoA, which is an essential biosynthetic precursor for lipid synthesis.^50^ In sheep, C1QTNF1 was significantly downregulated in the adipocytokine signaling pathway in the tail fat tissue of Altay sheep,^51^ and differentially expressed in the longissimus muscle tissue of Polled Dorset sheep.^52^ Owing to C1QTNF1 being positioned in the protein-coding region, further studies are needed to validate the association between citrate metabolism and the molecular function of C1QTNF. There are no specific biological functions annotated for the novel gene ENSOARG00020014135.

### Genome-wide association for the content of lactic acid

The enrichment analyses performed for the LA candidate genes uncovered four biological processes, with protein N-linked glycosylation via asparagine (GO:0018279) standing out as the most notable function. The specific functions of the most probable candidate gene for LA content, Cytohesin 1 (CYTH1), are related to the regulation of cell adhesion (GO:0030155), and regulation of ARF protein signal transduction (GO:0032012). CYTH1 could be involved in the regulation of casein synthesis (particularly through Glu) in bovine mammary epithelial cells.^53^^,^^54^

The second most probable candidate gene MGAT5B codes for protein N-linked glycosylation (GO:0006487) and protein O-linked glycosylation via serine (GO:0018242) processes. Interestingly, a study^55^ reported MGAT5B to be positively correlated with acetate, which is the first product of citrate metabolism and the intermediate of co-metabolism of glucose and citrate in milk.^41^^,^^55^ The co-metabolism of both glucose and citrate starts with the degradation of citrate into acetate and oxaloacetate, following that oxalacetate is catalyzed to pyruvate which is the intermediate for co-metabolism and an essential product of glucose metabolism.^56^ It has been identified that co-metabolism results in a decreased uptake of glucose and increased acetate and D-lactate production, in other metabolic functions; citrate had a slightly inhibitory effect on the enzymes of the ‘ethanol’ leg of glucose metabolism.^56^ The gene MGAT5B was found to be differentially expressed in Glycan biosynthesis and lipid metabolism processes in cervical cancer cells, regulating the decrease of lipid peroxidation, and the increase in glucose consumption.^57^ In dairy cattle milk, MGAT5B was associated with clinical mastitis and SCC.^58^

In dairy cows, TIMP2 is involved in the regulation of intra-mammary infections and fibrosis process.^59^ The gene MAZ participates in the regulation of calcium-dependent activation of synaptic vesicle fusion (GO:0150037), and synaptic vesicle fusion to presynaptic active zone membrane (GO:0031629). Also, it has been reported that the MAZ gene is a transcription factor that is highly upregulated in chronic inflammatory disease.^60^

The gene NUPR1 is associated with acute inflammatory response (GO:0002526), and negative regulation of glycolytic process (GO:0045820). Also, NUPR1 is related to the degradation of insulin storages and subsequent secretion during inflammatory and obesity-related tissue stress.^61^

The association between genes MAZ and NUPR1 and LA content in milk has not been identified in previous GWAS studies, but these genes are associated with mastitis in cows^62^ and buffalo.^63^ It has been reported that LA content can also be used as a signal molecule of the immune system and has novel signal transduction functions in preventing excessive inflammatory responses, both intracellular and extracellular.^64^^,^^65^ It has been suggested that LA content in milk is a strong indicator of mastitis in early lactating cows and goats, with a positive correlation between LA and SCC.^28–30^ The identified genes in this study can be useful for future GWAS related to sheep mastitis traits.

## Conclusions

High heritability was estimated for both LA and CA traits. This study was the first to report genomic variance for CA and LA, and a strong negative genetic correlation was found between CA and LA. The genomic background of these traits has not yet been investigated in dairy sheep and is only recently being investigated for dairy cows, which highlights the unique contribution of this study. Despite the small sample size, the genome-wide association analysis in our study detected a total of 9 SNPs and 56 candidate genes associated with CA and LA content. Of notable importance for both CA and LA contents in sheep milk were the genetic variations in chromosome 11. The functions of genes are associated with energy balance, metabolic processes, protein glycosylation, immunity response, and clinical mastitis, which may provide more information for research on LA and CA metabolism in sheep and on using LA and CA as metabolic biomarkers for energy balance and clinical mastitis. The findings of the present study lack robustness for large-scale applications due to the small sample size. Therefore, further genomic studies must be performed with larger populations of dairy sheep. Studies on gene expression could also validate the present results.

## Supplementary Material

Supplemental Material

## Data Availability

The data presented in this experiment are available within the article.
