# Supplementary material for: Genome-wide association studies for citric and lactic acids in dairy sheep milk in a New Zealand flock
Source: Anim Biotechnol. 2024 Aug 5;35(1):2379897. doi: 10.1080/10495398.2024.2379897 (PMC12674232; doi:10.1080/10495398.2024.2379897)
Supplement: Supplemental Material [file LABT_A_2379897_SM0488.docx]

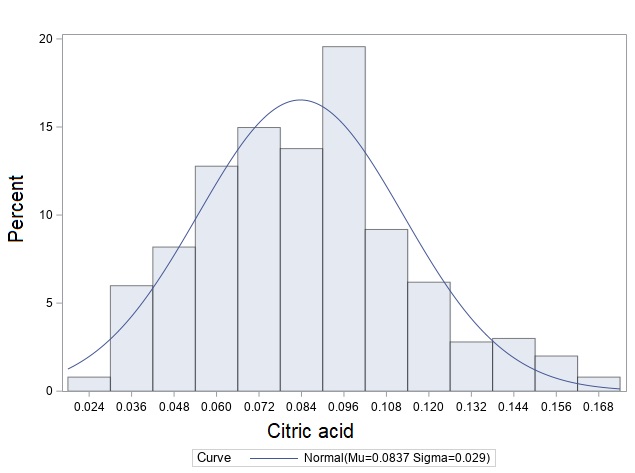


**Figure S1.** Data distribution plot for citric acid.


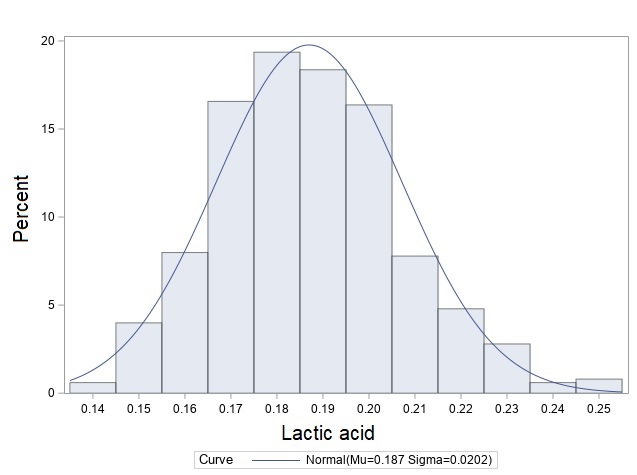


**Figure S2**. Data distribution plot for lactic acid.


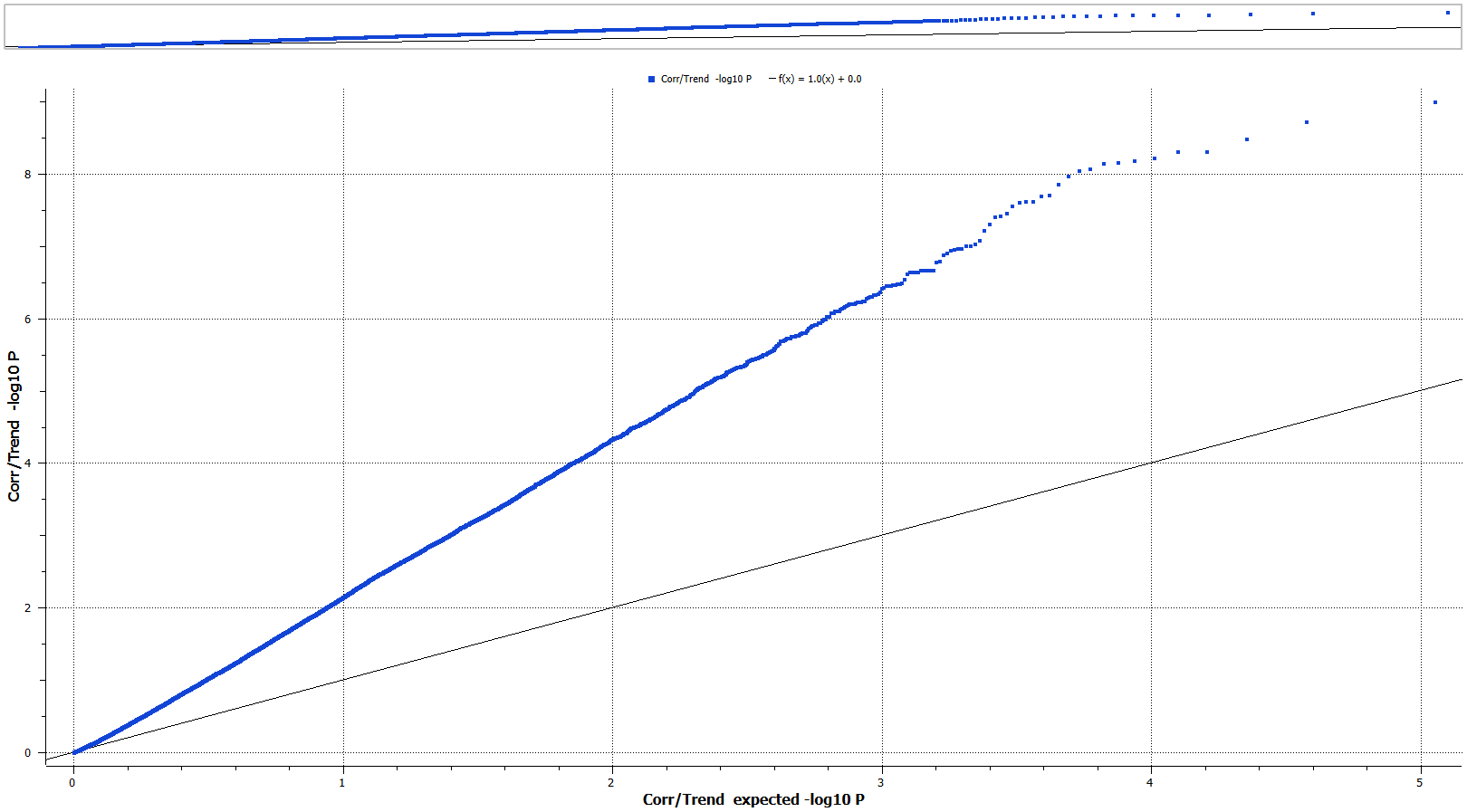


**Figure S3.** QQ-plot for citric acid raw data before correction for stratification.


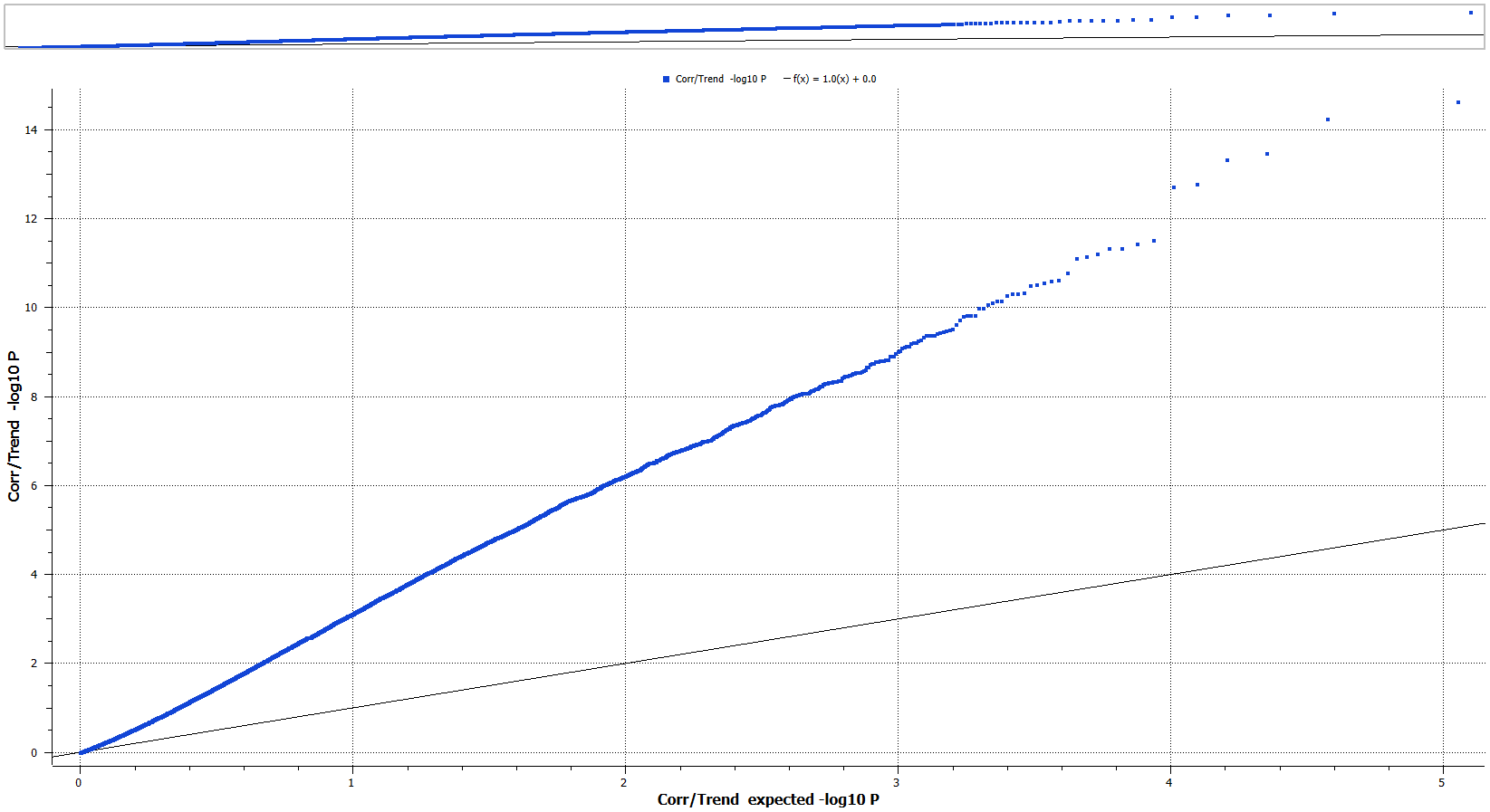


**Figure S4.** QQ-plot for lactic acid raw data before correction for stratification.


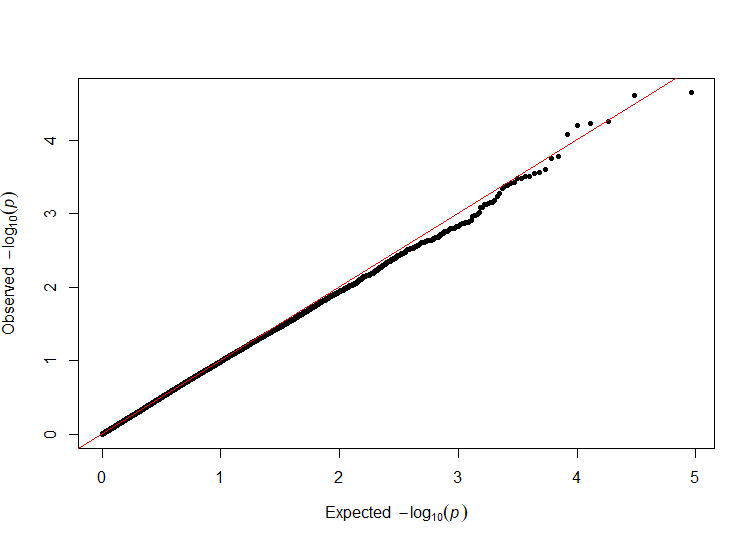


**Figure S5.** QQ-plot for citric acid data corrected for stratification.


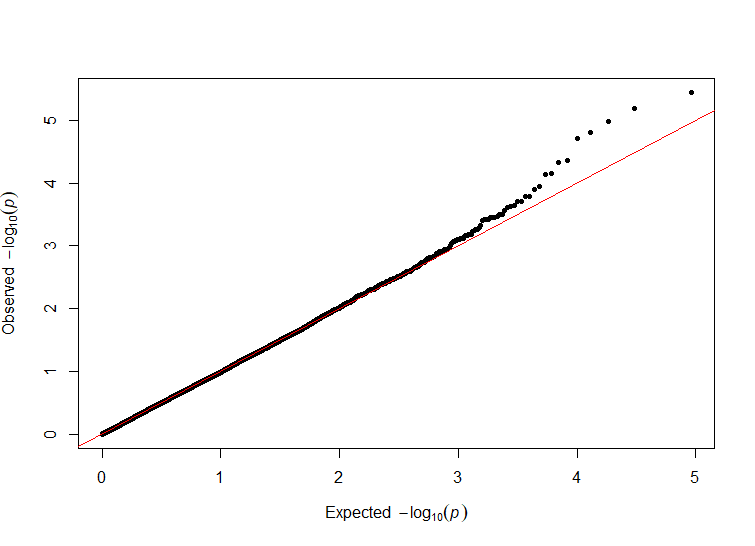


**Figure S6.** QQ-plot for lactic acid data corrected for stratification.
